# Clinical presentation, transmissibility, and virulence of mpox clades in Africa: a systematic review (2023–2025): Exploring clinical presentation, transmissibility, and virulence of mpox based on clades

**DOI:** 10.1186/s40794-026-00304-4

**Published:** 2026-05-19

**Authors:** Mosoka Papa Fallah, Abigael Abiy Mesfin, Shahd Osman Sayed Osman, John Nyagaka, Tinotenda Tavruinga, Patrick Chanda Kabwe, Maikem Viktorine, Farisai Kuonza, Manar Keshk, Tamrat Shaweno, Banda Khalifa

**Affiliations:** 1 Africa Centers for Disease Control and Prevention, Addis Ababa, Ethiopia; 2 University of Toronto, Toronto, Canada; 3 Johns Hopkins Bloomberg School of Public Health, Baltimore, MD USA

**Keywords:** Mpox, Clinical presentation, Virulence, Africa

## Abstract

**Background:**

Mpox is caused by mpox virus and comprises distinct clades with differing clinical and epidemiologic profiles. Clade I (Ia, Ib) predominates in Central Africa, and clade IIb drove the 2022–2023 global outbreak. This review assessed clinical presentation, transmissibility, and virulence of mpox in African settings by viral clade.

**Methods:**

Searches of PubMed/MEDLINE, PubMed Central, Scopus, Cochrane Library, and Google Scholar identified studies from January 2023 to May 2025. Titles and abstracts were screened in Rayyan by two reviewers, with conflicts resolved by a third. Literature search was conducted between July to August 2025. Eligible designs included cohort studies, case series, case reports, and surveillance reports with clade assignment and clinical or transmission data. Risk of bias was appraised with the JBI tools. Data were synthesized narratively. PRISMA guidelines were followed.

**Results:**

Ten studies from the Democratic Republic of Congo, Burundi, Uganda, Kenya, and Nigeria met inclusion criteria. Four were cohort designs and six were case reports or case series. Sample sizes ranged from 1 to 850. Reported clades were Ia, Ib, and clade I unspecified; several reports also described clade IIb-linked contexts. Rash was the most consistent symptom, reported in 80–100% of cases. Reported transmission routes included sexual contact, close non-sexual contact, household exposure, travel-related exposure, and vertical transmission confirmed by placental PCR in two studies. Adverse outcomes were frequent in pregnancy, with fetal loss reported in 50–67% in small series. Deaths occurred among people with untreated or advanced HIV infection.

**Conclusion:**

The reviewed evidence further affirms the dominant role of sexual transmission in current African outbreaks, contrasting with earlier assumptions of predominantly zoonotic or household spread. Strengthening surveillance systems that can distinguish mpox clades and track their clinical patterns is essential to guiding effective public health responses.

**Supplementary Information:**

The online version contains supplementary material available at 10.1186/s40794-026-00304-4.

## Introduction

Mpox, previously known as monkeypox, is caused by the mpox virus (MPXV), a double-stranded DNA virus in the *Orthopoxvirus* genus. It has been endemic in Central and West Africa, with most human cases resulting from zoonotic spillovers in the rainforests of the Democratic Republic of the Congo (DRC) and nearby countries, with limited human-to-human transmission [1, 2]. In recent years, the epidemiology of mpox in Africa has changed significantly. Sustained outbreaks have been driven by distinct viral clades, increased human-to-human transmission, and growing public health concerns. By 2023, the DRC reported 14,626 suspected cases, including 654 deaths, and again in 2024, about 12,569 cases with 581 fatalities were reported [3]. Additionally, by mid-2024, a surge of mpox cases across multiple countries in Central Africa prompted the World Health Organization (WHO) and the Africa Centres for Disease Control (Africa CDC) to declare a Public Health Emergency of International Concern [4–7]. 

The mpox virus (MPXV) comes in two varieties: clade I and clade II, each of which has two subclades (Ia, Ib, IIa, IIb). Similar methods can be used to prevent, treat, and disseminate both clades and all subclades [8]. The viral clades have different epidemiological and clinical characteristics. Clade I, historically dominant in Central Africa, is split into clades Ia and Ib. Clade Ia is mainly linked to zoonotic transmission and outbreaks in rural children, presenting with high fever, lymphadenopathy, and extensive rashes. It has historically had case-fatality rates up to 10% and can lead to severe complications like encephalitis and pneumonia [9, 10]. Clade Ib, first identified in South Kivu Province of the DRC in 2023, shows enhanced human-to-human transmission, especially through sexual contact, often resulting in localized genital lesions [10, 11]. While it appears to be less severe than clade Ia, its rapid spread in urban areas has raised concerns about its epidemic potential [12] Clade IIb, first identified in West Africa, had limited human-to-human transmission until 2017. An outbreak in Nigeria from 2017 to 2021 resulted in several months of urban transmission, leading to the unprecedented global mpox outbreak of 2022–2023 driven by lineage B.1 [13]. Many clade IIb cases presented atypically, with mild or absent prodromal symptoms, localized anogenital lesions, and significant mucosal involvement. While the overall case fatality rate was low in healthy adults, severe disease and fatalities occurred among those with untreated HIV, accompanied by high hospitalization rates [10, 14].

Recently, the emergence of the G.1 lineage (A.2.2.1), a descendant of clade IIb, has led to significant outbreaks in Sierra Leone and neighboring countries since early 2025. The virus is spreading rapidly within both household and intimate-contact networks, affecting women and children, which highlights the growing public health significance of this lineage [15]. However, previous researches [16–18] have mainly examined individual outbreaks. While characterization of the disease by clades is important, there is limited comparative analysis across clades in Africa, where the disease burden is highest. This systematic review evaluated the clinical presentation, transmissibility, and virulence of mpox clades 1a, 1b, and 2b. The findings will provide crucial insights for clinical management and public health interventions in Africa. This review aims to systematically synthesise the available evidence on the epidemiologic characteristics, clinical presentation, transmissibility, and virulence of mpox specified across different viral clades.

## Methodology

A comprehensive search was performed in the following electronic databases; PubMed/MEDLINE, PMC, Google Scholar, Scopus, and Cochrane Library. We designed the search strategy using Medical Subject Headings (MeSH) terms and keywords such as ‘monkeypox’ ‘mpox’, ‘epidemiology’, ‘transmission’, ‘Clade I’, ‘Clade II’, ‘clinical features’, ‘Symptoms’, ‘presentation’, ‘manifestation’. Mixed mesh terms were used to search for articles. ((“Monkeypox“[MeSH] OR monkeypox OR mpox) AND (transmissibility OR transmission OR “disease transmission”) AND (virulence OR severity OR pathogenicity) AND (clade OR lineage OR variant OR “genetic variation”) AND (Africa OR African)). This review included only primary studies reporting original data on the clinical presentation, transmissibility, and virulence of mpox stratified by viral cladein Africa published between 2023 up to May 2025. No language restrictions were applied during the search. The final database search was conducted on June 30, 2025. The search strategy was designed to identify primary studies reporting on the clinical presentation, transmissibility, and virulence of mpox stratified by viral clade, in line with PRISMA reporting recommendations. The full search strategies for each database, including all keywords and Boolean operators, are provided in Supplementary Material 1.

### Study selection

391 articles were retrieved from Pubmed Central, 55 from Pubmed, 33 from Scopus, 1from Cochrane library and 669 from Google Scholar. A total of 1149 were retrieved from and imported to Rayyan. Duplicates were identified on Rayyan and resolved by taking the author, the year of publication, the article title, and the volume, issue and number of pages into consideration. After duplicates were removed, 746 articles remained. Two reviewers independently screened titles and abstracts against the predefined eligibility criteria, followed by full-text screening of potentially relevant articles. A standardized screening form, developed based on the inclusion and exclusion criteria, was used to ensure consistency in study selection. Inter-reviewer agreement was assessed during the screening process, and any discrepancies between reviewers were resolved through discussion and consensus. Where consensus could not be reached, a third reviewer was consulted to make the final decision. Screening of articles took place from July 1, 2025 to August 1, 2025. Finally 15, articles fulfilled the criteria from title and abstract screening. Subsequently, full text screening was done and 10 articles qualified for data extraction (Fig. 1).

### Eligibility criteria

The following inclusion and exclusion criteria were used.

### Inclusion criteria

- Studies conducted either in endemic or non-endemic regions and will provide data on disease incidence, geographical distribution, demographic characteristics, transmission dynamics, clade characteristics and clinical outcomes.
- Studies reporting human mpox cases with confirmed clade classification (Clade I or II).
- Studies presenting data on clinical signs, symptoms, severity, complications, or outcomes.
- Peer-reviewed articles, case reports, case series, surveillance reports, cohort and cross-sectional studies.
- Studies published from 2023 to May 2025.

### Exclusion criteria

- Animal-only studies.
- Opinion pieces, editorials, commentaries, and conference abstracts without primary data.
- Review articles including literature, narrative review and systematic reviews.
- In vitro or genomic-only studies without clinical data.
- Studies without clade-specific data or where clade distinction cannot be made.
- Papers for suspected cases.
- Parts of a book without primary data.

### Data extraction

A standardized data extraction form was developed. Extracted data included:

- Study characteristics (author, year, country, study design).
- Population characteristics (age, sex, number of cases).
- Clade type (Clade Ia, b or Clade II, a, b).
- Clinical features (rash type and distribution, fever, lymphadenopathy, systemic symptoms).
- Disease severity and complications (e.g., hospitalization, encephalitis, secondary infections).
- Outcomes (recovery, mortality).

### Quality assessment

Two independent reviewers (Reviewer A and Reviewer B) assessed each study for risk of bias. Discrepancies were resolved through discussion, and when necessary, a third reviewer was consulted to reach consensus. Risk of bias was assessed through JBI checklist. Each item within the checklist was scored as 1 (“Yes”) or 0 (“No/Unclear”), and a total score was calculated for each study. Studies scoring 7–9 were rated as low risk, 4–6 as moderate and < 4 as high risk. The domains assessed included selection of participants, measurement of exposure and outcomes, identification and management of confounding factors, and completeness of reporting. The quality assessment indicated that a substantial proportion of studies were classified as having moderate to high risk of bias. Evidence derived from studies with lower scores was interpreted with caution.

The included studies demonstrated substantial heterogeneity in study design, sample size, and methodological approaches. These ranged from single case reports and small case series to larger observational cohort studies. This variability influenced the level of detail and robustness of reported outcomes, particularly for clinical severity, complication rates, and transmission patterns. As such, findings should be interpreted with caution, as differences across studies may reflect methodological variation as well as true differences between study populations and settings.

### Data synthesis

We presented the findings using qualitative approach through narrative synthesis.

## Results

### Flow of included studies

**Fig. 1:**
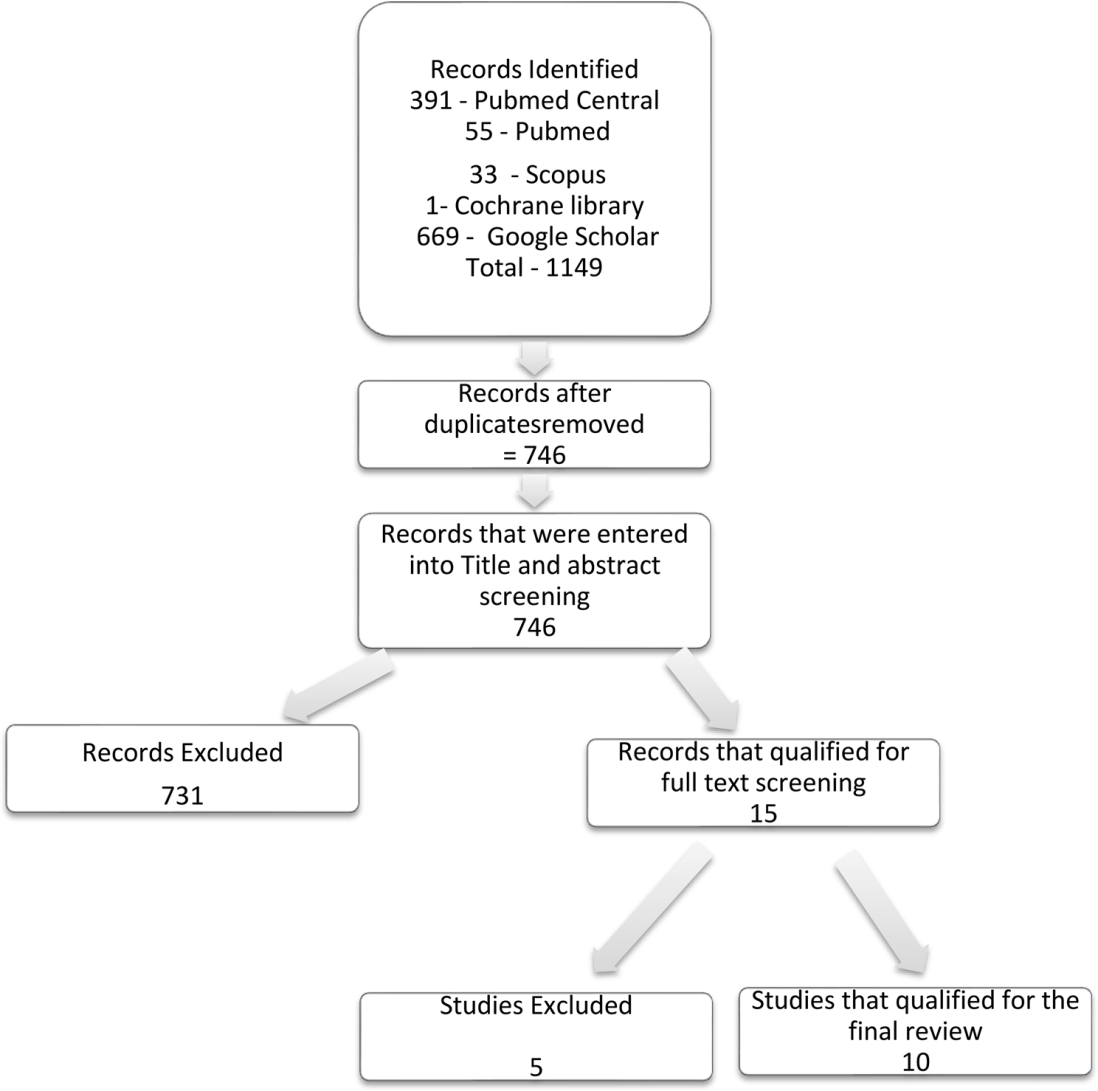
PRISMA flowchart for article screening

### Study characteristics

We included 10 studies published between 2023 and 2025 that provided data on clinical presentation, transmissibility, severity, and special populations (pregnant women and people living with HIV) of Mpox across different clades (Table 1). Studies originated from multiple African countries including the Democratic Republic of Congo (DRC), Burundi, Uganda, Kenya, and Nigeria.

Of the included studies, four were cohort studies (two prospective, one retrospective cross-sectional, and one open cohort), while the remaining were case reports or case series. The sample size across studies ranged from single-case reports to large cohorts, with the largest cohort comprising 850 individuals [19]. The clades of mpox virus identified in these studies included Clade Ia, Clade Ib, and Clade I (subclade unspecified).

Studies demonstrated varied age distributions, covering a wide spectrum from young infants (as young as 3 months old) to adults (up to 71 years old). There was generally balanced representation of male and female patients, although certain smaller case series and case reports were exclusively female.

### Clinical presentation

The clinical presentation of mpox varied substantially across the different clades included in our review (Table 2). Rash emerged as the most consistently reported symptom, observed in 80% to 100% of cases across most studies, affirming its role as a hallmark feature. Fever was also frequently reported but showed greater variability, ranging from as low as 5% in the Brosius et al. ([3], Clade Ib, DRC) cohort to as high as 100% in individual case reports (Makangara-Cigoloet al., 2025, Clade Ia, DRC) [20].

Lymphadenopathy presented with notable variability across studies. It was universally reported (100%) in a small case report by Bbosa et al. (2025, Clade Ib, Uganda) [21], while markedly lower prevalence rates were documented in other studies (14.6% by Pius Mutuku et al., 2025, Clade Ib, Kenya) [22] and absent in certain cases (0% reported by Makangara-Cigolo et al., 2025, Clade Ia, DRC) [20]. These variations might be attributable to differences in clinical presentations by clade, as well as demographic or geographical factors.

Other frequently reported clinical symptoms included genital lesions, particularly prevalent among patients with Clade Ib infections. Fatigue, myalgia, headache, oral lesions, anorectal lesions, and respiratory distress were also commonly documented symptoms, although prevalence varied widely across studies.

### Transmissibility

The modes of transmission identified in our review included sexual transmission, close contact (non-sexual), vertical transmission (mother-to-child), and household exposure (Table 3). Sexual transmission was commonly reported in most studies, including those by Brosius et al. ([3], Clade Ib), Makangara-Cigolo et al. (2025, Clade Ia), Nkengurutse et al. (2025, Clade Ib) [19], Cibenda et al. (2025, Clade I) [23], Schwartz et al. (2024, Clade I) [24], Pius Mutuku et al. (2025, Clade Ib) [22], Kibungu et al. (2024, Clade I) , and Masirika et al. (2023–24, Clade Ib) [25], suggesting this may be a predominant mode of mpox transmission in African contexts.

Vertical transmission was documented in studies by Brosius et al. [3] and Masirika et al. (2023–24) [25], who specifically confirmed the presence of mpox virus in placental tissue by PCR. Non-sexual close contact, including household contact, was widely reported across several studies [19, 23, 26], reflecting the significant risk of intrafamilial transmission. However, these findings are largely derived from case reports and case series, which may not fully represent population-level transmission dynamics.

Notably, Kibungu et al. (2024) [27] reported a secondary attack rate of 7.6%, providing quantitative evidence of human-to-human transmission dynamics within household settings, although such detailed transmission data were not reported by other studies.

### Disease severity and virulence

Disease severity varied widely among the included studies, with reports ranging from mild, self-limiting infections to severe, complicated disease with hospitalization and mortality (Table 4). Severe disease was consistently observed in most studies, with hospitalization rates ranging from 2.1% (Pius Mutuku et al., 2025, Clade Ib, Kenya) [22] to as high as 100% in multiple studies ([3]; 19, 23 Masirika et al., 2023–24) [25]. This high hospitalization rate reflects both the severity of clinical presentations and possibly the threshold for admission in various health-care contexts.

Significant complications were reported across multiple studies, including severe genito-urinary symptoms (57%) and cutaneous manifestations (41%) reported by Brosius et al. [3]. Nkengurutse et al. (2025, Clade Ib) [19] documented serious genital complications such as vaginitis, genital ulceration, and complicated pyelonephritis, with rare occurrences of Fournier gangrene. Furthermore, Cibenda et al. (2025, Clade I) [23] observed severe dermatitis (69.4%) and anemia (37.9%) as prominent complications, highlighting diverse clinical manifestations across clades.

Mortality rates varied significantly, with some studies reporting no deaths [19, 27] and others reporting mortality as high as 7 deaths (Masirika et al., 2023–24, Clade Ib) [25]. Notably, Makangara-Cigolo et al. (2025, Clade Ia) [20] documented a single fatal case complicated by malaria co-infection, emphasizing the potential role of comorbid conditions in determining disease outcomes.

These findings underscore the complexity and severity of mpoxinfections across different clades and emphasize the importance of prompt clinical intervention, particularly in populations with severe disease presentations and complications.

### Special populations (pregnancy and people living with HIV)

Adverse outcomes among special populations, including pregnant women and people living with HIV, were prominently documented in multiple studies (Table 5). Pregnant women with mpox faced high rates of adverse outcomes, notably miscarriages, stillbirths, and fetal losses. Schwartz et al. ([24], Clade I) reported a fetal loss rate of 50% among affected pregnant women, while Brosius et al. ([3], Clade Ib) documented adverse pregnancy outcomes in 67% of pregnant women. Similarly, Masirika et al. (2023–2024, Clade Ib) [25] documented fetal losses in 8 out of 14 affected pregnancies (57%), underscoring the significant risk mpox poses during pregnancy.

Delivery by cesarean section was specifically reported by Bbosa et al. (2025, Clade Ib, Uganda) [21], suggesting possible concerns related to transmission risks during childbirth.

Among people living with HIV, severe outcomes were evident. Pius Mutuku et al. (2025, Clade Ib, Kenya) [22] reported the death of one patient with advanced HIV disease not receiving antiretroviral therapy (ART), highlighting the role of underlying HIV status in disease severity.

Similarly, Masirika et al. (2023–2024, Clade Ib) [25] documented the death of all three people living with HIV identified in their study, further emphasizing increased mortality risk in this vulnerable group.

### Quality assessment and publication bias

While most studies in our dataset are peer-reviewed case series or outbreak reports emphasizing severe or high-transmission outcomes, at least one preprint is included (‘First Imported Cases of MPXV Clade Ib in Goma, DRC’). This suggests some gray literature was captured, although it remains a minority. Quality was assessed using JBI checklist. 2 articles had low risk of bias, 7 had moderate risk of bias and 1 had high risk of bias (Table 6).

**Table 1:** Study characteristics of included studies

Author	Year	Country	Study Design	Number of cases	Age Distribution	Sex % M/F *n* M/F	Clade Type
Brosius et al.	2025	DRC	Prospective Cohort	407	Bimodal(< 5, 15–34)	52/48 211/196	Ib
Jean-Claude Makangara-Cigolo et al.	2025	DRC	Case Report	1	> 20	0/100 0/1	Ia
David A. Schwartz et al.	2024	DRC	Case Series	8	15–29	0/100 0/8	Clade I(subclade Unspecified)
Nkengurutse et al.	2025	Burundi	Prospective Cohort	850	3 months–71 years	54.4/ 45.6 462/388	Ib
Cibenda et al.	2025	DRC	Open cohort	343	11–28	58.6, 41.4 201/142	Clade I(subclade Unspecified)
Bbosa et al.	2025	Uganda	Case Report	2	22&37	0/100 0/2	Ib
Pius Mutuku et al.	2025	Kenya	Case Series	48	IQR: 29–38	41.7/58.3 20/28	Ib
Kibungu et al.	2024	DRC	Case Series	5	26–35	75/25 3 / 1 (1 unspecified)	Clade I(subclade Unspecified)
Mukadi-Bamuleka et al.	2024	DRC	Case Series	9	6–45	(44.4%/55.6%) 4/9	Ib
Masirika et al.	2023-24	DRC	Retrospective Cross-sectional	646	15–24 (majority)	(47.6%/52.4%) 319/351	Ib

**Table 2:** Clinical presentation of mpox by study and clade type

Author	Year	Clade Type	Fever (%/*n*)	Rash (%/*n*)	Lymphadenopathy (%/*n*)	Other Significant Symptoms
Brosius et al.	2025	Ib	5%/ 20	97%/ 395	73%/ 297	77.4% (315) had genital lesions, fatigue 91% (370), itching 93% (379), malaise 77% (313), pain associated with lesions 79% (321), myalgia 73% (297), headache 60% (244), anorexia 58% (236), throat ache or dysphagia 56% (228), cough 36% (146), abdominal pain 31% (126), nausea or vomiting 23% (94), shortness of breath 14% (57), diarrhea 13% (53), and confusion 3% (12)
Jean-Claude Makangara-Cigolo et al.	2025	Ia	100%/ 1	100%/ 1	0%/ 0	Fever, arthralgia, later respiratory distress
David A. Schwartz et al.	2024	Clade I(subclade Unspecified)	N/A	12.5%/ 1 (Skin lesions on the macerated fetus)	N/A	N/A
Nkengurutse et al.	2025	Ib	54.5%/463	85.9%/730	33.3%/ 283	Genital lesions (46.9%/ 399), oral lesions (27.5/234), anorectal lesions (21.3%/181), headache (31.6%/269), fatigue (35.1%/298)
Cibenda et al.	2025	Clade I(subclade Unspecified)	75.2%/258	100%/343	23.6%/ 81	Genital skin lesions (43.1%/148)
Bbosa et al.	2025	Ib	50%/ 1	100%/ 2	100%/ 2	
Pius Mutuku et al.	2025	Ib	85.4%/ 41	100%/ 48	14.6%/ 48	Genital lesions (68.7%/33), Headache (41.7%/20)
Kibungu et al.	2024	Clade I(subclade Unspecified)	60%/ 3	80%/4	N/A	Genital Lesions (80%/ 4)
Mukadi-Bamuleka et al.	2024	Ib	66.7%/32	100%/9	77.8%/37	Genital Lesions (77.8%/ 37), Oral lesions (66.7%/32)
Masirika et al.	2023-24	Ib	N/A	N/A	N/A	1 infant – Born with intrauterine skin lesion

N/A – Data not available

**Table 3:** Transmissibility of mpox by study and clade type

Author	Year	Clade Type	Mode of Transmission	Secondary Attack Rate (%)
Brosius et al.	2025	Ib	Sexual transmission, Vertical transmission,	N/A
Jean-Claude Makangara-Cigolo et al.	2025	Ia	Sexual Transmission	N/A
David A. Schwartz et al.	2024	Clade I(subclade Unspecified)	Close contact, Sexual Transmission, Travel	N/A
Nkengurutse et al.	2025	Ib	Sexual Contact, Household exposure	N/A
Cibenda et al.	2025	Clade I(subclade Unspecified)	Sexual Contact, Close Contact	N/A
Bbosa et al.	2025	Ib	Close Contact, Travel	N/A
Pius Mutuku et al.	2025	Ib	Sexual Transmission, Travel, Close Contact	N/A
Kibungu et al.	2024	Clade I(subclade Unspecified)	Sexual Transmission, Household Contact	7.6%
Mukadi-Bamuleka et al.	2024	Ib	Close Contact(Non-sexual)	
Masirika et al.	2023-24	Ib	Sexual transmission Vertical transmission(PCR positive placenta was found)	N/A

N/A – Data not available

**Table 4:** Disease severity and virulence of mpox by study and clade type

Author	Year	Clade Type	Severity (Mild, Severe)	Hospitalization (%/*n*)	Major Complications(%/*n*)	Mortality (*n*)
Brosius et al.	2025	Ib	50%/ 203 Severe	100%/407	Genito-urinary(57%/232), cutaneous (41%/ 167)	2
Jean-Claude Makangara-Cigolo et al.	2025	Ia	100% Severe(1 case)	100%/1	Malaria (co- infection)	1 death
David A. Schwartz et al.	2024	Clade I(subclade Unspecified)	50% Severe	12.5%/1	Fetal loss	0 death among pregnant women (4 fetal loss)
Nkengurutse et al.	2025	Ib	Mild (66.6%/ 566) Moderate (21.2%/180), Severe (12.2%/104)	100%/ 850	Vaginitis (1.1%/9), genital ulceration (0.5%/4), complicated pyelonephritis with vaginitis (0.1%/1) and Fournier gangrene (0.1%/1)	0
Cibenda et al.	2025	Clade I(subclade Unspecified)	Severe (21%/72)	100%/343	Dermatitis (69.4%/ 238), Anemia (37.9%/130), skin abscesses (13.7%/47)	2 death
Bbosa et al.	2025	Ib	Mild	N/A	Resolved	0
Pius Mutuku et al.	2025	Ib	N/A	2.1%/ 1(Data not mentioned for the rest of confirmed cases who were isolates either at homes or health facilities, No specific information)	2.1%/1 ((With Cryptococcal meningitis, Secondary bacterial infections of lesions, Severe constipation) 1 stillbirth	1 death
Kibungu et al.	2024	Clade I(subclade Unspecified)	Mild to Moderate	0%	N/A	0 death
Mukadi-Bamuleka et al.	2024	Ib	N/A	N/A	N/A	N/A
Masirika et al.	2023-24	Ib	N/A	100%/646	0.15% /1Neurological complications and Respiratory distress, 57%(8), Intrauterine fetal death among pregnant	7 deaths

N/A – Data not available

**Table 5:** Special populations (pregnancy and people living with HIV)

Author	Year	Clade Type	Pregnant women (*n*)	Pregnancy Outcomes clearly Described(%)	People living with HIV (*n*)	Outcomes of patients with HIV clearly described
Brosius et al.	2025	Ib	21	67% adverse outcomes	6	6
Jean-Claude Makangara-Cigolo et al.	2025	Ia	0	Not Applicable	Discordant Result(Tested both positive and negative for HIV)	N/A
David A. Schwartz et al.	2024	Clade I(subclade Unspecified)	8	50% fetal loss	1	N/A
Nkengurutse et al.	2025	Ib	7	N/A	28	N/A
Cibenda et al.	2025	Clade I(subclade Unspecified)	10	N/A	9	N/A
Bbosa et al.	2024	Ib	1	Delivery By CS	N/A	N/A
Pius Mutuku et al.	2025	Ib	1	Still born at 38 weeks	11	One patient died, He had advanced HIV disease and was not on ART
Kibungu et al.	2024	Clade I(subclade Unspecified)	0	N/A	N/A	N/A
Mukadi-Bamuleka et al.	2024	Ib	0	N/A	0	N/A
Masirika et al.	2023-24	Ib	14	8 fetal loss	3 confirmed/ Not screened for all	3 of the confirmed died

N/A – Data not available

**Table 6:** Quality score of articles

Author	Year	Study Design	JBI Score
Brosius et al.	2025	Prospective Cohort	5
Jean-Claude Makangara-Cigolo et al.	2025	Case Report	3
David A. Schwartz et al.	2024	Case Series	4
Nkengurutse et al.	2025	Prospective Cohort	5
Cibenda et al.	2025	Open cohort	5
Bbosa et al.	2024	Case Report	6
Pius Mutuku et al.	2025	Case Series	7
Kibungu et al.	2024	Case Series	6
Mukadi-Bamuleka et al.	2024	Case Series	8
Masirika et al.	2023-24	Retrospective Cross-sectional	9

## Discussion

Our findings highlight substantial heterogeneity in the clinical presentation of mpox across different viral clades, consistent with emerging literature on the re-emergence and geographic spread of Clade Ia and Ib variants in Africa. Rash was universally reported as the most consistent symptom, present in 80–100% of cases across nearly all reviewed studies. This aligns with classical mpox case descriptions from historical outbreaks, where rash was a cardinal sign irrespective of clade or setting [1, 2]. Rash has retained its diagnostic significance in both Clade Ia and Clade Ib contexts. Fever, while also common, showed marked variation from as as 5% ([3], Clade Ib, DRC) to 96% [4]. Such variability may stem from clade-specific immune responses or differing definitions of fever onset across studies. Notably, other literature, such as Yinka-Ogunleye et al. [5] in Nigeria and Thornhill et al. [6], a global outbreak, have similarly reported fever rates between 60% and 85%, indicating variability even outside central African contexts.

Lymphadenopathy emerged as a particularly heterogeneous symptom. While 100% prevalence was observed in the Ugandan study (Bbosa et al., 2024), it was significantly lower in Kenya (14.6%, Pius Mutuku et al., 2025, Clade Ib) [22] and entirely absent in a Clade Ia cohort (Makangara-Cigolo et al., 2025, DRC) [20]. These findings mirror prior reports indicating that Clade Ib may be more lymphotropic than Clade Ia [5, 7]. The absence of lymphadenopathy in Clade Ia cases may also be linked to milder systemic inflammation or delayed presentation. Genital lesions were disproportionately observed in Clade Ib cases, consistent with sexual transmission pathways increasingly reported during recent outbreaks. Thornhill et al. [6] observed genital lesions in 70% of patients in a multicenter global cohort, which aligns with findings from our Clade Ib cases. Similarly, Ogoina et al. [8] described sexual transmission among MSM populations in Nigeria, with localized lesions predominating in the anogenital region. Fatigue, myalgia, and headache were variably reported. For example, headaches were noted in 45% to 75% of Clade Ib cases in Kenya and Uganda, similar to previous findings by Ejvar JJ et al. [9] in a US outbreak. Such symptoms, while non-specific, reinforce the importance of a syndromic approach during surveillance. Oral and respiratory symptoms (e.g., oral lesions, dyspnea) were occasionally reported and may reflect either more severe disease or co-infections. This is supported by Satapathy et al. [10] in which respiratory symptoms were reported in 20–30% of mpox cases.

Concerning the transmissibility of mpox across Clades and settings, the findings from our review underscore the heterogeneity of mpox transmission pathways, shaped by clade-specific virological characteristics, behavioral factors, and contextual epidemiological settings. The major modes of transmission included sexual contact, close (non-sexual) contact, vertical transmission, and household exposure, each varying in prominence across studies. Sexual transmission was a consistently reported mode in both Clade Ia and Clade Ib studies across multiple countries [3, 19, 20, 22, 23, 27]. These findings align with global outbreak patterns since 2022, particularly in Clade IIb contexts, where sexual networks, especially among MSM, have played a central role in transmission [11]. Importantly, our review affirms that sexual transmission is also dominant in African Clade I settings, contrasting with earlier views that primarily emphasized zoonotic or household routes. While sexual transmission was previously underreported in endemic countries due to stigma or lack of sexual history documentation, emerging evidence now points to mucocutaneous lesions in genital and perianal regions and PCR-positive semen samples as indicators of direct sexual transmission potential (CDC, 2023). Vertical transmission, long suspected but rarely confirmed, was substantiated in our review by molecular detection of mpox virus in placental tissue in the studies by Brosius et al. [3] and Masirika et al. (2023–24) [25]. These cases provide compelling support for in utero fetal infection, which mirrors findings from Mbala et al. [13], who described fetal demise and placental mpox DNA detection in the DRC.WHO and CDC have recognized vertical transmission as a plausible but poorly documented route ([14]; CDC, 2023), and its confirmation in multiple Clade I and Ib cases in our review suggests a potentially higher burden of adverse pregnancy outcomes in endemic areas. This contrasts with Clade IIb outbreaks in high-income settings, where few if any vertical transmission cases have been documented [24]. The household transmission context was quantitatively analyzed in the study by Kibungu et al. (2024) [27], which reported a secondary attack rate of 7.6%, in line with prior estimates ranging between 3 and 11% in DRC [17]. Such transmission is influenced by housing density, care-seeking behavior, and hygiene practices, which differ markedly between urban and rural African settings and may not reflect dynamics in recent Clade IIb outbreaks in the West.

Although several studies in this review highlight sexual contact as a commonly reported exposure, caution is warranted in interpreting this as the dominant mode of transmission, particularly within African contexts. Much of the available evidence is derived from case reports and small case series, which are inherently subject to selection and reporting biases. These study designs may overrepresent specific transmission routes, especially those that are more easily identifiable or of particular clinical interest. In addition, incomplete exposure histories and social desirability bias may further limit the accurate characterization of transmission pathways. Therefore, while sexual transmission appears to play an important role in certain settings, the current evidence base is insufficient to definitively establish it as the predominant mode of transmission at the population level. More robust epidemiological studies with systematic exposure assessment are needed to better define transmission dynamics.

The severity of mpox disease in our review ranged from mild, self-limiting illness to severe disease with complications, hospitalization, and mortality, reflecting a pattern observed in both Clade I and Clade Ib infections. Hospitalization rates in our review varied widely from 2.1% (Pius Mutuku et al., 2025, Clade Ib, Kenya) [22] to 100% in Brosius et al., Nkengurutse et al., Cibenda et al., and Masirika et al. This variation likely reflects not only disease severity but also differences in admission thresholds, availability of outpatient follow-up, and public health response levels. These findings are consistent with previous reports from endemic regions, where Clade I is often associated with higher hospitalization and complication rates than Clade II strains [18]. In contrast, recent Clade IIb outbreaks (e.g., [6, 28]) observed lower hospitalization rates (< 10%), often limited to supportive care, suggesting lower virulence or more benign clinical courses in those settings.

Severe complications especially genital lesions, cutaneous damage, and hematological abnormalities were frequently observed in Clade Ib and Clade I cases. Brosius et al. [3] reported genitourinary complications in 57% of patients, while Cibenda et al. (2025) [23] found severe dermatitis in 69.4% and anemia in 37.9%. These complications were often debilitating and required intensive medical care. Similar findings were reported by Ogoina et al. [29] in Nigeria (Clade Ib), where up to 25% of hospitalized patients developed complications such as secondary bacterial infections, ocular lesions, or bronchopneumonia. In contrast, Clade IIb outbreaks largely affecting MSM communities have typically featured localized anogenital lesions, with fewer systemic complications [6, 30]. Mortality in the reviewed studies ranged from zero (Nkengurutse et al., Schwartz et al., Bbosa et al., Kibungu et al.) to as high as 7 deaths in Masirika et al. (2023–24, Clade Ib) [25]. Makangara-Cigolo et al. (2025, Clade Ia) [20] reported a fatal case complicated by malaria co-infection, emphasizing how coinfections and comorbidities can exacerbate outcomes, echoing historical DRC studies. Comparatively, mortality in Clade IIb outbreaks has been extremely low (< 0.1%), with most fatalities occurring in immunocompromised individuals (e.g., persons with HIV/AIDS) [31]. In contrast, Clade I historically had case fatality rates as high as 10.6% [7], supporting the idea that clade-specific virulence plays a central role in disease outcomes.

Our review reveals that mpox infection in special populations particularly pregnant women and individuals living with HIV is associated with markedly worse clinical outcomes, including high rates of fetal loss, severe complications, and mortality. Multiple studies in our review report a high burden of fetal complications among pregnant women with mpox. Schwartz et al. [24], examining Clade I infections, reported fetal loss in 50% of infected pregnant women. Similarly, Brosius et al. [3] found adverse pregnancy outcomes in 67% of Clade Ib cases. In another study by Masirika et al. (2023–2024) [25], fetal loss was observed in 57% (8 out of 14) of pregnancies affected by Clade Ib. These findings strongly align with historical reports from the DRC. For instance, Mbala et al. [13] reported intrauterine fetal demise and placental mpox DNA positivity, confirming vertical transmission and poor perinatal outcomes. In addition, earlier Clade I–associated mpox outbreaks in endemic regions documented serious pregnancy complications, including fetal loss and congenital infection.

For instance, a prospective cohort study in the Democratic Republic of the Congo between 2007 and 2011 reported that 3 out of 4 pregnant women with laboratory‑confirmed Clade I mpox experienced fetal death, and viral infection was demonstrated in placental and fetal tissues [32]. During a 2023–2024 Clade I outbreak in South Kivu Province, DRC, eight pregnant women were studied, of whom four (50%) had fetal losses, reaffirming the high risk of adverse obstetric outcomes with Clade I infection [24]. In contrast, pregnancy-related outcomes in Clade IIb outbreaks have been less frequently reported, largely due to the underrepresentation of pregnant women in outbreak cohorts. However, isolated reports (e.g., [24] have raised concerns about potential fetal risks, even in Clade IIb, though fetal demise and miscarriage rates have been lower in those settings. The documentation of caesarean deliveries (e.g., Bbosa et al., 2024) suggests concerns over intrapartum transmission, mirroring practices in other viral infections (e.g., herpes simplex virus) and reflecting the lack of standardized delivery guidelines for mpox-positive pregnancies.

Our review indicates that people living with HIV, particularly those with advanced or untreated infection, face a markedly increased risk of severe mpox disease and death. For instance, Pius Mutuku et al. (2025, Kenya) [22] reported a fatality in a patient with advanced, untreated HIV, while Masirika et al. (2023–2024, DRC) [25] documented 100% mortality (3 out of 3 cases) among HIV-positive individuals in their cohort. These findings are consistent with global data on Clade IIb mpox. Patel et al. [31] and Philpott et al. [28] observed that severe, protracted illness and fatalities were concentrated among individuals with low CD4 counts or without access to antiretroviral therapy (ART). Similarly, Thornhill et al. [6] and Ogoina et al. [29] reported more extensive lesions, complications, and hospitalizations among people living with HIV, especially in Clade Ib and IIb contexts. Notably, well-controlled HIV infection defined by ART use and normal CD4 counts has not been consistently linked to worse outcomes. These observations underscore the critical role of early ART initiation and the need to integrate HIV care into mpox prevention and treatment strategies.

This review has several limitations that should be acknowledged. First, the heterogeneity in study design, population characteristics, and clinical definitions across the included studies limits the comparability of findings and may affect the consistency of disease severity and outcome measures. Second, reporting biases, including underreporting of mild cases and limited laboratory confirmation in some settings, may have influenced the observed distribution of clinical severity and complications. Third, data on special populations such as pregnant women and people living with HIVwere often limited in scope and size, restricting the generalizability of findings. Additionally, most studies lacked long-term follow-up, making it difficult to assess the full spectrum of disease progression and recovery. Finally, geographic clustering of studies in a few countries may limit the applicability of these findings to other endemic or emerging regions.

## Conclusion

This review underscores the complex and heterogeneous clinical and epidemiological profile of mpox across Clade I and Clade Ib variants in African settings. Rash remains a defining symptom, while other clinical features including fever, lymphadenopathy, and genital lesions demonstrated variability linked to viral clade, geography, and population demographics. The reviewed evidence further affirms the dominant role of sexual transmission in current African outbreaks, contrasting with earlier assumptions of predominantly zoonotic or household spread. Vertical transmission and severe disease manifestations, including hospitalization, complications, and mortality, were most prominent in Clade I and Ib infections, especially among pregnant women and people living with HIV. These findings align with historical data from the Democratic Republic of the Congo and contrast with recent Clade IIb outbreaks in high-income settings, where disease courses have been generally milder.

Strengthening surveillance systems that can distinguish mpox clades and track their clinical patterns is essential to guiding effective public health responses. Risk communication should incorporate sexual health messaging, given the predominance of sexual transmission in recent outbreaks. Special attention must be given to pregnant women and people living with HIV, who are at higher risk of adverse outcomes, by ensuring timely diagnosis, targeted clinical care, and preventive measures. Health systems should also be prepared to manage severe and complicated mpox cases through improved hospital readiness. Equitable access to vaccines, therapeutics, and research tailored to vulnerable populations remains critical to reducing disease burden and improving outcomes. Strengthening diagnostic capacity is also fundamental. Finally, data gaps should be addressed through more evidence synthesis conducted in non-DRC endemic regions.

## Electronic Supplementary Material

Below is the link to the electronic supplementary material.

Supplementary Material 1

Supplementary Material 2

## Data Availability

Data extraction sheets are available from the corresponding author upon request.
