# Supplementary material for: Clinical presentation, transmissibility, and virulence of mpox clades in Africa: a systematic review (2023–2025): Exploring clinical presentation, transmissibility, and virulence of mpox based on clades
Source: Trop Dis Travel Med Vaccines. 2026 May 19;12:20. doi: 10.1186/s40794-026-00304-4 (PMC13188601; doi:10.1186/s40794-026-00304-4)
Supplement: Supplementary file 1 — Supplementary Material 1 [file 40794_2026_304_MOESM1_ESM.docx]

**Full Search Strategy**

**1. Pubmed and PMC**

("Monkeypox"[MeSH] OR monkeypox OR mpox) AND (transmissibility OR transmission OR "disease transmission") AND (virulence OR severity OR pathogenicity OR complications OR "clinical manifestation" OR "clinical manifestations") AND (clade OR lineage OR variant OR "genetic variation")

Mesh Terms

| Concept | MeSH Term | Additional Keywords (Free Text) |
| --- | --- | --- |
| Monkeypox | "Monkeypox"[Mesh] | mpox, "monkeypox virus", "monkey pox" |
| Epidemiology | "Epidemiology"[Subheading]OR "Epidemiology"[Mesh] | outbreak, distribution, incidence, prevalence |
| Transmission | "Disease Transmission, Infectious"[Mesh] | transmission, spread, contact, "sexual transmission" |
| Clade I / II | *(No specific MeSH)* | "Clade I", "Clade II", "Congo Basin clade", "West African clade" |
| Clinical Features | "Signs and Symptoms"[Mesh]OR "Clinical Presentation"[Mesh] | "clinical features", "presentation", "manifestation" |
| Symptoms | "Signs and Symptoms"[Mesh] | symptoms, rash, fever, lymphadenopathy |

("Monkeypox"[Mesh] OR mpox OR "monkeypox virus" OR "monkey pox")

AND

("Disease Transmission, Infectious"[Mesh] OR transmission OR spread OR contact OR "sexual transmission")

AND

("Signs and Symptoms"[Mesh] OR "Clinical Presentation"[Mesh] OR symptoms OR "clinical features" OR presentation OR manifestation)

AND

("Clade I" OR "Clade II" OR "West African clade" OR "Congo Basin clade")

This MeSH term was used.

**2. Cochrane**

Epidemiology AND Transmission, Virulence, Clade Variations AND Clinical Features of AND Monkeypox (Mpox)

**3. For SCOPUS**

TITLE-ABS-KEY("monkeypox" OR "mpox" OR "monkeypox virus" OR "MPXV")

AND

TITLE-ABS-KEY("epidemiology" OR "transmission" OR "clade I" OR "clade II" OR "clinical features" OR "symptoms" OR "presentation" OR "manifestation")

**4. For Google Scholar**

("monkeypox" OR "mpox" OR "monkeypox virus" OR "MPXV") AND ("clade I" OR "clade II") AND ("clinical features" OR "clinical manifestations" OR "symptoms" OR "presentation" OR "signs" OR "manifestation") AND ("epidemiology" OR "transmission" OR "incidence" OR "spread" OR "outbreak") AND ("Virulence") AND ("Human")
